# Biosynthesized Multivalent Lacritin Peptides Stimulate Exosome Production in Human Corneal Epithelium

**DOI:** 10.3390/ijms21176157

**Published:** 2020-08-26

**Authors:** Changrim Lee, Maria C. Edman, Gordon W. Laurie, Sarah F. Hamm-Alvarez, J. Andrew MacKay

**Affiliations:** 1 Department of Pharmacology and Pharmaceutical Sciences, School of Pharmacy, University of Southern California, Los Angeles, CA 90033, USA; changril@usc.edu; 2 Department of Ophthalmology, USC Roski Eye Institute and Keck School of Medicine, University of Southern California, Los Angeles, CA 90033, USA; edman@usc.edu; 3 Department of Cell Biology, School of Medicine, University of Virginia, Charlottesville, VA 22908, USA; gwl6s@virginia.edu; 4 Department of Biomedical Engineering, Viterbi School of Engineering, University of Southern California, Los Angeles, CA 90089, USA

**Keywords:** lacritin, peptide biosynthesis, corneal epithelium, exosome, syndecan-1, elastin-like polypeptides

## Abstract

Lacripep is a therapeutic peptide derived from the human tear protein, Lacritin. Lacripep interacts with syndecan-1 and induces mitogenesis upon the removal of heparan sulfates (HS) that are attached at the extracellular domain of syndecan-1. The presence of HS is a prerequisite for the syndecan-1 clustering that stimulates exosome biogenesis and release. Therefore, syndecan-1-mediated mitogenesis versus HS-mediated exosome biogenesis are assumed to be mutually exclusive. This study introduces a biosynthesized fusion between Lacripep and an elastin-like polypeptide named LP-A96, and evaluates its activity on cell motility enhancement versus exosome biogenesis. LP-A96 activates both downstream pathways in a dose-dependent manner. HCE-T cells at high confluence treated with 1 μM LP-A96 enhanced cell motility equipotent to Lacripep. However, cells at low density treated with 1 μM LP-A96 generated a 210-fold higher number of exosomes compared to those treated at low density with Lacripep. As monovalent Lacripep is capable of enhancing cell motility but not exosome biogenesis, activation of exosome biogenesis by LP-A96 not only suggests its utility as a novel molecular tool to study the Lacritin biology in the corneal epithelium but also implies activity as a potential therapeutic peptide that can further improve ocular surface health through the induction of exosomes.

## 1. Introduction

The lacrimal gland-corneal axis plays a critical role in maintaining ocular health. The lacrimal gland is the major organ responsible for the secretion of essential proteins and electrolytes into the tear film that overlays and protects the cornea and conjunctiva [1]. One of these essential tear proteins that confer anti-microbial and anti-inflammatory defense at the ocular surface is Lacritin [2]. A 12.3 kDa secreted glycoprotein found in human and non-human primates, Lacritin exhibits prosecretory activity in lacrimal gland acinar cells (LGAC) and mitogenic activity in corneal epithelial cells [3]. Originally discovered by the Laurie laboratory [3], several studies have revealed that the active monomeric form of Lacritin is significantly downregulated in patients suffering from chronic blepharitis [4], aqueous-deficient dry eye [5], contact-lens related dry eye [6], and dry eyes associated with primary Sjögren’s syndrome (SS) [7]. It remains unclear if Lacritin monomer down-regulation (in part through tissue transglutaminase-dependent cross-linking at the syndecan-1 binding domain [8]) is a symptom or a direct cause of these ocular surface diseases; however, Lacritin has shown potential as a therapeutic molecule. Its supplementation enhanced tear secretion from rat LGAC [3] and monkey LGAC [9], increased basal tear secretion in rabbit LGAC [10] and dry eye mouse eyes [11], and stimulated human corneal epithelial (HCE-T) cell proliferation [12]. This composite of preclinical evidences supports the continued development of Lacritin as an ocular therapeutic. Currently, its peptide derivative, Lacripep^TM^, is under clinical evaluation for protein replacement therapy for dry eye disease (DED) and SS-associated DED (NCT03226444).

Syndecan-1 present in corneal epithelium is a known receptor for Lacritin. The cleavage of heparan sulfate (HS) chains at the extracellular domain of Syndecan-1 by an enzyme called heparanase enables Lacritin binding to syndecan-1 [13]. Heparanase exists in inactive (proheparanase) and active (heparanase) forms. Each of these forms has been shown to engage with syndecan-1 and to mediate distinct downstream signaling events. Heparanase cleaves HS and promotes association of Lacritin and syndecan-1 [14]. This interaction promotes cell proliferation and increases the motility of corneal epithelial cells by activating PKCα and NFAT-related pathways [12]. Proheparanase can cluster syndecan-1 by binding to intact HS chains attached to syndecan-1 [15,16,17]. The consequent clustering and internalization of syndecan-1 is known to activate the exosome biogenesis pathway by recruiting related molecules, including ALG-2-interacting protein X (ALIX), syntenin, and endosomal-sorting complex required for transport (ESCRTs). Therefore, HS-mediated activation of mitogenic signaling (requiring removal of HS from syndecan-1) and exosome biogenesis (requiring attachment of HS to syndecan-1) in the corneal epithelium are assumed to be mutually exclusive.

This study explores a newly generated self-assembled multivalent Lacritin peptide nanoparticle named LP-A96, that may activate both pathways in corneal epithelial cells. Specifically, 1 μM LP-A96 enhanced cell motility at a high cell density, whereas its ability to promote exosome biogenesis was more prominent at a low cell density. The enhancement of cell motility by LP-A96 at 1 μM was equipotent to that of the monovalent Lacripep but its ability to induce exosome biogenesis was markedly higher, enabling the production of 210-fold higher number of exosomes in a given period of time compared to 1 μM Lacripep and 58-fold higher number of exosomes compared to the complete medium. Both actions of LP-A96 occurred in parallel with mobilization of intracellular Ca^2+^ and syndecan-1 internalization. With increasing interest in Lacritin and in exosome biology at the ocular surface, this study serves as a foundation for understanding ocular surface homeostasis, pathophysiology, and possible therapeutic interventions.

## 2. Results

### 2.1. Biosynthesized LP-A96 Fusion Proteins Self-Assemble into Multivalent Nanoparticles

For clarity, ‘LP’ refers to the lacritin-derived peptide used to generate LP-A96, and ‘Lacripep’ refers to the peptide equivalent to the one currently in clinical trials (NCT03226444). Both the LP and Lacripep used in this study are derived from the active fragment at the C-terminus of the human Lacritin: GKQFIENGSEFAQKLLKKFSLLKPWA. While both LP and Lacripep share an identical 19 amino acid sequence as a core (Table 1), LP contains five additional amino acids: the N-terminal methionine encoded by the start codon followed by a glycine (MG), which is found in human lacritin. LP also contains at the C-terminus, a leucine-tryptophan-alanine (LWA). During the optimization of expression, three amino acids, leucine-lysine-proline (LKP), were removed because this region was prone to autocleavage. This cleavage was reported previously [18] and reconfirmed by an independent mass analysis (data not shown). The WA, which is also found at the next two amino acids of the endogenous Lacritin protein, was included at the C-terminus of the 19-mer core to aid in the spectroscopic estimation of concentration due to the high absorbance of the included tryptophan. Although the LP contains several additional amino acids compared to Lacripep, the 19-mer core that exhibits activity remained unaltered. The regions that contain those five additional amino acids were confirmed to have no activity related to syndecan-1 binding and mitogenic activity exhibited by the 19-mer core [12].

**Figure 1:**
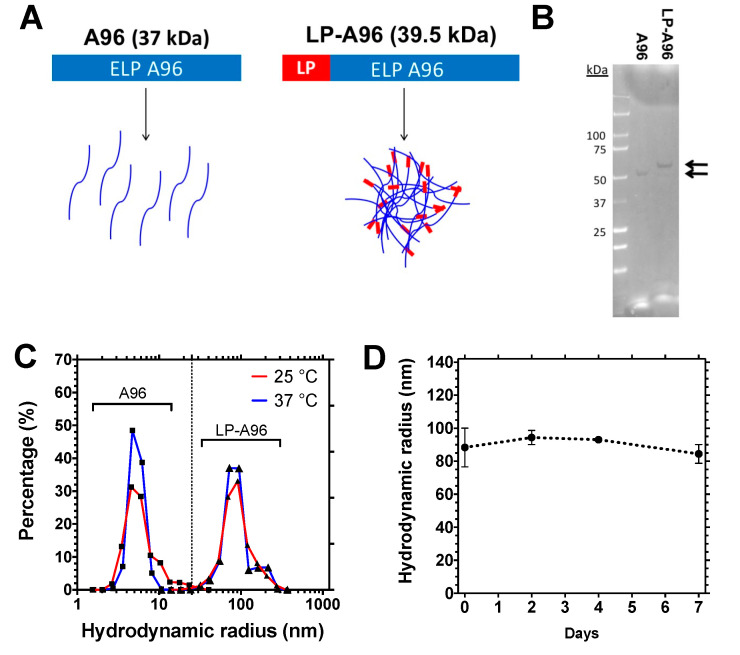
Lacripep promotes assembly of LP-A96 into stable, multivalent spherical nanoparticles. (**A**) Design of LP-A96 fusion. Through heterologous expression in *Escherichia coli*, the LP was fused to the N-terminus of the elastin-like polypeptide (ELP), A96. (**B**) The identity and purity of A96 and LP-A96 were analyzed by Coomassie blue staining of samples resolved by SDS-PAGE (sodium dodecyl sulfate-polyacrylamide gel electrophoresis). Arrows indicate protein bands. A 20–30% upward shift in MW (molecular weight) with respect to the expected MW for ELPs and ELP fusions in SDS-PAGE has been observed by us and others [25,26]. The exact MW of LP-A96 was measured using MALDI-TOF-MS (Appendix A Figure A1D). (**C**) Comparison of the hydrodynamic radius of monomeric A96 and self-assembled LP-A96 nanoparticles at room and physiological temperatures. (**D**) The hydrodynamic radius of LP-A96 remains stable at 37 °C for least 7 days in DPBS (10 μM, *n* = 3, mean ± SD).

**Figure A1:**
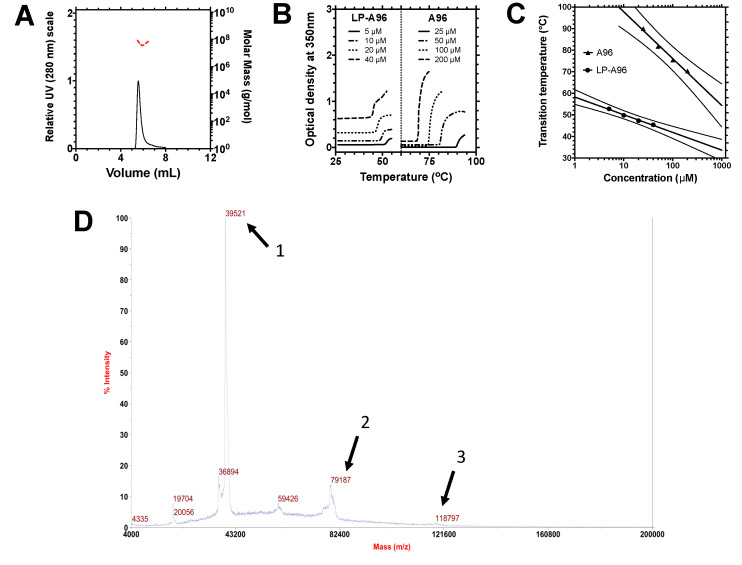
Biophysical characteristics of LP-A96. (**A**) LP-A96 elutes from size exclusion chromatography followed by multiangle light scattering (SEC-MALS) as a single peak (solid line according to left *Y*-axis) consistent with a nanoparticle with a radius of gyration of 79.3 nm (red dotted line according to the right *Y*-axis). (**B**) The ELP-mediated phase separation is monitored using optical density at 350 nm as a function of temperature using UV–Vis spectrophotometry (10 μM in dPBS). ELP phase separation increases the optical density. Transition temperatures were identified using Equation (4). (**C**) A log-linear relationship between transition temperature and concentration was fit using Equation (3). Parameters of the fit are reported in Table A1. Dotted lines indicate 95% CI. (**D**) MALDI-TOF-MS analysis of LP-A96. The molecular weight of 39,521 Da (black arrow 1) is consistent with expected molecular weight reported in Table 1. Minor species identified at 79,187 Da (black arrow 2) and 118,797 Da (black arrow 3) are consistent with the masses expected for transglutaminase-mediated dimers and timers of LP-A96 [8].

The 23-mer LP was genetically fused to the N-terminus of an elastin-like polypeptide (ELP) termed A96 (Table 1). Emerging as an attractive recombinant protein–polymer choice for diverse applications, ELPs are under exploration as a drug delivery platform since their biosynthesis produces pharmacologically relevant, monodisperse, biodegradable, and biocompatible entities [20,21,22]. The sequence confirmed cDNA encoding the LP-A96 fusion protein was subjected to heterologous expression via bacterial fermentation. The yield after the purification was ~30 mg/L with >95% purity, as verified by SDS-PAGE (sodium dodecyl sulfate-polyacrylamide gel electrophoresis) (Figure 1A,B). To determine the hydrodynamic radius (*R_h_*) and colloidal stability, purified LP-A96 was analyzed using dynamic light scattering (DLS) at days 0, 2, 4, and 7 (Figure 1C,D). Unlike A96 alone, the 23-mer LP induces assembly of LP-A96 into a colloidally stable nanoparticle, which persists even below its *T_t_*. Purified LP-A96 remained stable for at least 7 days at 37 °C with average *R_h_* of 90.1 nm (±7.2 nm, SD). Size-exclusion chromatography followed by multi-angle light scattering (SEC-MALS) showed that all LP-A96 monomers self-assembled to nanoparticles (Appendix A Figure A1A). The radius of gyration (*R_g_*) was 79.3 nm (± 0.6%, SEM). The estimated shape factor (ratio between *R_g_* and *R_h_*) of 0.88 (spheres ≈ 0.8 < *R_g_*/*R_h_* < 2.4 ≈ rods) suggests that the LP-A96 particles are spherical [23,24].

Given the thermo-responsive nature of ELPs, the optical density of an LP-A96 solution was scanned at 350 nm (OD 350) over a range of temperatures (Appendix A Figure A1B) to determine its phase transition temperature (*T_t_*). ELPs or ELP nanoparticles become insoluble microparticles (in a process called phase separation or coacervation) that can be measured by an increase in the OD 350 [27]. Based on the generated phase diagram (Appendix A Figure A1C), LP-A96 nanoparticles are expected to remain stable colloids at physiological temperatures and not phase-separate at the concentrations used in this study.

### 2.2. LP-A96 Induces Ca^2+^ Mobilization, Enhances Cell Motility, and Stimulates Exosome Biogenesis in Corneal Epithelial Cells

The central hypothesis of this study is that, because of its multivalent nanoparticulate nature relative to Lacripep, LP-A96 may have the ability to elicit pathways that enhance cell motility and stimulates exosome biogenesis in corneal epithelial cells. To test this, we started by exploring the consequences of LP-A96 treatment in HCE-T cells on cell motility and Ca^2+^ mobilization.

**Figure 2:**
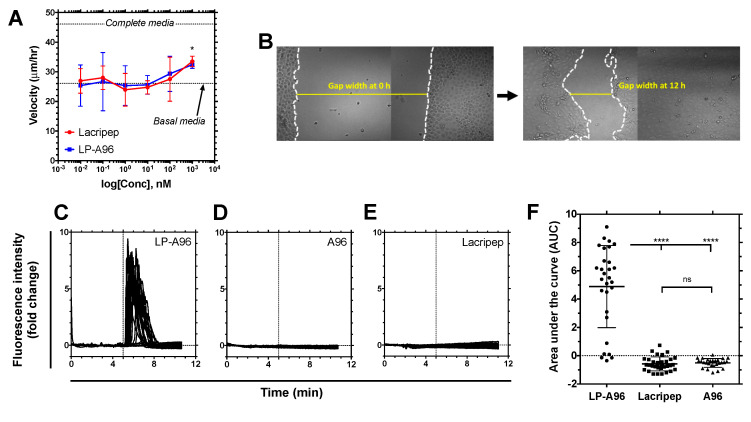
LP-A96 promotes scratch closure and induces Ca^2+^ influx in corneal epithelial cells. (**A**) A scratch (gap) assay was used to observe the enhancement of cell motility. The velocity of cell monolayer towards the remaining gap area (devoid of cells) was calculated as outlined in (**B**). (**B**) Gaps were imaged before the treatment and at 12 h post-treatment, and used to construct (**A**). White dotted lines indicate the outer edges of the monolayer adjacent to the wound gap. (**C~E**) Human corneal epithelial (HCE-T) cells pre-incubated with Fluo-4 AM were incubated with either LP-A96, Lacripep, or A96 (all at 1 μM). The dotted line at the 5th minute indicates when the treatment was added to the culture medium. The fluorescence intensity of Fluo-4 AM was monitored as a marker for Ca^2+^ influx (*n* = 27~33 cells/treatment per experiment). A representative data set from the three independent sets of the experiment is shown. (**F**) The area under the curve of each fluorescence intensity profile shown in (**C**~**E**) showed that only LP-A96 extensively mobilized Ca^2+^. A one-way ANOVA followed by multiple comparisons was used for statistical comparison. **** *p* < 0.0001, * *p* < 0.05, ns: non-significant. Mean ± SD.

Both LP-A96 and Lacripep enhanced cell motility at a dose of 1 uM in a cell monolayer scratch assay (Figure 2A,B). Lacritin-mediated cell motility enhancement in corneal epithelial cells has been associated with Ca^2+^ mobilization [12]. It has been shown that extracellular Ca^2+^ translocates into the cytoplasm (influx), where it interacts with calcineurin and subsequently activates downstream signaling related to cell motility. To measure Ca^2+^ influx, fluorescence intensity changes in individual cells incubated with Fluo-4AM were monitored by fluorescence microscopy in real-time upon addition of LP-A96 to culture media. LP-A96 induced a strong Ca^2+^ influx immediately after the addition (Figure 2C,F). As there was no increase in fluorescence intensity upon addition of A96 (Figure 2D), it was apparent that the LP component of LP-A96 was essential for inducing Ca^2+^ influx. However, under identical conditions, Lacripep did not induce Ca^2+^ influx (Figure 2E).

Although Ca^2+^ influx has previously been linked to lacritin-mediated cell motility, the degree of Ca^2+^ influx required for cell motility alone may be below the limits of detection in this assay. If this is the case, the superior Ca^2+^ influx induced by LP-A96 may represent evidence of activation of another pathway that Lacripep does not activate. One possibility in the context of syndecan-1 biology would be an activation of the exosome biogenesis pathway, which also requires Ca^2+^ influx [15,28].

**Figure 3:**
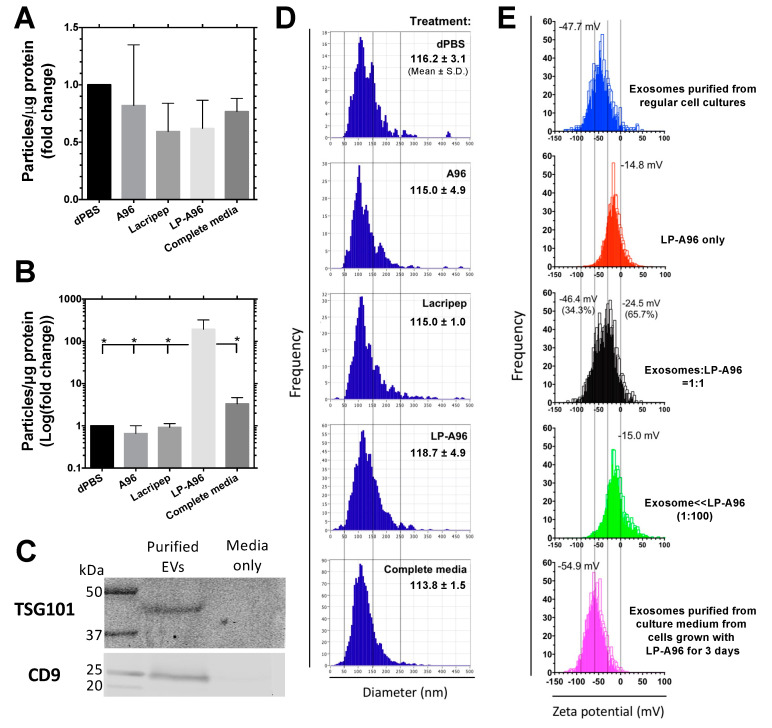
LP-A96 activates exosome biogenesis in corneal epithelial cells. (**A**) Nanoparticle tracking analysis was used to quantify extracellular vesicles (EVs) in the collected HCE-T cell culture media under each treatment at near maximum confluency. Cells cultured to 80~90% confluency were starved for another 24 h using basal media prior to the treatment. EVs in the culture media were purified after 3 days of treatment. (**B**) EVs in the collected HCE-T cell culture media under each treatment at subconfluency (30%). Cells were supplemented with different agents upon seeding (0.5 × 10^5^ cells/well in 12-well plate). EVs in the culture media were purified after 3 days of seeding. The raw increase in EV amount upon LP-A96 treatment was corrected based on the Western blot-based analysis (Appendix A Figure A2) before being analyzed for fold change. For (**A**,**B**), all treatments were at 1 μM. (**C**) EVs collected after LP-A96 treatment were enriched in the exosome markers CD9 and TSG101. (**D**) Bar graphs show the size distribution of purified exosomes. The three parallel gray lines indicate 50, 150, and 250 nm, respectively. Representative data from three independent sets of the experiment is shown. Inserted numbers indicate mean hydrodynamic diameter of purified exosomes. (**E**) Zeta potential analyses of exosomes, LP-A96, and combinations of both at different ratios. The four parallel gray lines indicate −90, −60, −30, and 0 mV, respectively. Inset values are the mean zeta potentials reported by the built-in software. A one-way ANOVA followed by multiple comparisons was used for statistical comparison. * *p* < 0.05. Mean ± SD.

To observe whether LP-A96 promotes exosome biogenesis, extracellular vesicles (EVs) secreted into the culture media over a three-day period were collected and analyzed. Exosome biogenesis was not prominent when cells were at a high confluence (Figure 3A). However, at subconfluence, cells incubated with LP-A96 spawned a significantly higher number of EVs (Figure 3B) that were highly enriched with exosome markers [29] (Figure 3C). The number of EVs collected from the culture media from cells after LP-A96 treatment was 210-fold and 58-fold higher compared to the amount recovered in basal media supplemented with Lacripep and in complete media, respectively. The mean diameter and the size distribution of the purified EVs were similar among groups (Figure 3D). To observe the rate of exosome biogenesis/release, exosomes were collected at 36 and 72 h post-LP-A96 treatment. The total exosome number and exosome biogenesis/release rate during 36 h were found to be significantly different from those during 72 h. This suggests the rate of exosome uptake/turnover exceeds the rate of exosome biogenesis/release during the 36~72 h period (Appendix A Figure A4).

To confirm that this apparent increase in exosome amount was not due to residual LP-A96 co-purified during the purification, purified exosomes were subjected to zeta potential analysis. Firstly, the zeta potential of the natural exosomes collected from regular cell cultures (blue bar graph in Figure 3E) and the preparation of pure LP-A96 (red bar graph in Figure 3E) were compared, which showed marked differences. Secondly, assuming that the residual LP-A96 in the culture media could be co-purified, two mixtures were prepared with different ratios: exosome:LP-A96 at 1:1 and 1:100. These mixtures were incubated at 37 °C for three days in basal media, subjected to the identical column-based purification process and then analyzed for zeta potential. For the 1:1 mixture, instead of a clear separation of two peaks, the profile was broadly distributed between −60~0 mV, exactly overlapping the profiles of pure exosome and LP-A96. Two zeta potential values were reported for this mixture: −46.4 mV and −24.5 mV (black bar graph in Figure 3E). For the 1:100 mixture, the highest peak was observed at around −15 mV and the profile was narrowly distributed between −30~0 mV, which highly resembled that of the pure LP-A96. Lastly, the zeta potential of exosomes purified after LP-A96 treatment was compared with the above four profiles. The mean value and the overall distribution profile of purified exosomes after LP-A96 treatment (magenta bar graph in Figure 3E) highly resembled those of the natural exosomes (blue bar graph in Figure 3E). Its mean zeta potential was −55 mV and the major portion of the profile was narrowly distributed between −90~−30 mV (−48 mV and −80~−20 mV, respectively, for natural exosomes). Therefore, it was apparent that the purified EVs after LP-A96 treatment are dominated by exosomes, and not LP-A96 particles.

**Figure A2:**
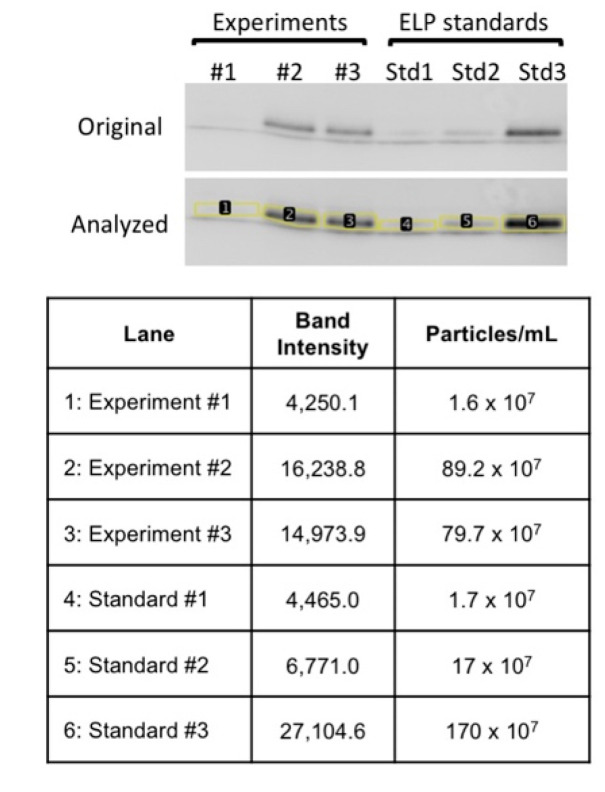
Western blot-based densitometric analysis for residual LP-A96 in purified EV samples. Purified EVs from three independent experiments (Experiments #1~3, lane 1~3) were blotted for ELPs using anti-ELP antibody AK1 [30] to detect and quantify residual LP-A96 co-purified with exosomes. To determine this, firstly, particle concentration of pure LP-A96 was determined (particle number/mL) using nanoparticle tracking analyzer. Secondly, serial dilutions of LP-A96 were prepared and their particle concentration was correlated to the band intensity in Western blot (ELP Standard #1~3, lane 4~6). Lastly, the standard curve generated based on this correlation was used to extrapolate the LP-A96 particle amount co-purified in EV samples after LP-A96 treatment (lane 1~3). This calculation was used to reflect the raw increase in exosomes in Figure 3B.

To identify the presence of any residual LP-A96 particles that were not detected during the zeta potential analysis, collected exosomes (used to construct Figure 3A and magenta bar graph in Figure 3E) were analyzed by Western blotting to detect ELPs using an anti-ELP antibody [30] along with a pre-determined amount of LP-A96 (Appendix A Figure A2). LP-A96 nanoparticles co-purified with the increased exosome yield were detected at low abundance as estimated by Western blotting, comprising about 10% of the total particles purified. Based on this densitometric analysis, the pure increase in exosome yield (210-fold and 58-fold increase compared to Lacripep and complete media treatment, respectively) associated with LP-A96 treatment was corrected (Figure 3A). Before correction, it was about 235-fold and 68-fold, respectively.

**Figure 4:**
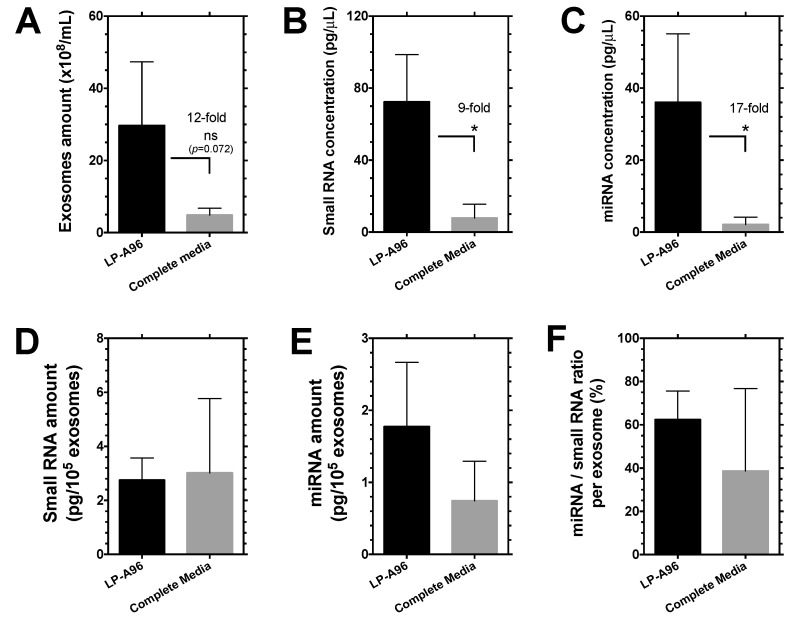
LP-A96 induced exosomes are properly loaded with RNAs. (**A**) Exosomes purified from subconfluent HCE-T cells after 36 h of incubation with either 1 μM LP-A96 in basal media or complete media shows stimulated exosome biogenesis and release by LP-A96 treatment. These exosomes were subjected to RNA isolation. (**B**,**C**) Elevated small RNA (0~270 nucleotides) and miRNA (10~40 nucleotides) amount reflects an increase in total exosome amount shown in (**A**). (**D**) Small RNAs, (**E**) miRNAs, and (**F**) miRNA-to-small RNA ratio per exosome particle under two different treatment conditions are similar. *n* = 3. Mean ± SD. * *p* < 0.05, ns: non-significant.

Exosomes mediate intercellular signaling. Their cargos, especially ribonucleic acids (RNAs), are important mediators for intercellular communication [31]. As LP-A96 stimulates exosome production, it was of interest to determine whether this important exosomal cargo was increased proportionally to the exosome number increase. During a 36 h incubation, cells treated with LP-A96 produced about 12-fold higher numbers of exosomes compared to cells exposed to complete media (Figure 4A). This increase in exosome abundance was correlated with a commensurate increase in both the total yield of non-coding small RNAs (9-fold) and miRNAs (17-fold) (Figure 4B,C). miRNAs are a subset of non-coding small RNAs often enriched in exosomes. When content was normalized to particle number, the amount of exosomal non-coding small RNAs and miRNAs per exosome particle was similar between groups (Figure 4D,E). About 60% of exosomal small RNAs were miRNAs in exosomes stimulated by LP-A96, which was slightly greater than that of exosomes stimulated by complete media, but there was no statistically significant difference (Figure 4F). This indicates that LP-A96 treatment produces exosomes that are likely functional and loaded with the proper amount of cargo RNA, similar to those produced under physiological growth condition, and not ‘blank’ exosomes.

**Figure 5:**
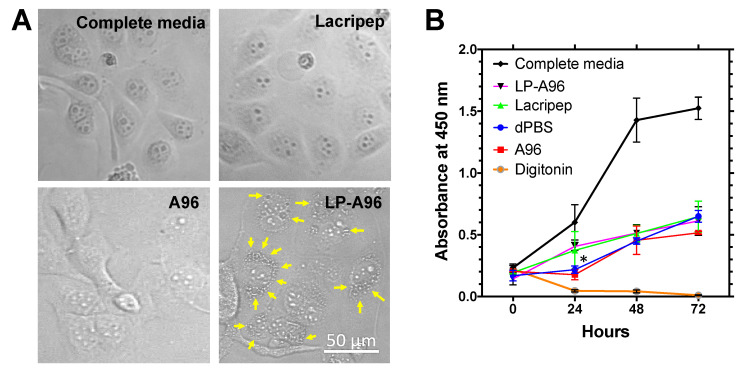
LP-A96-mediated exosome biogenesis does not affect cell viability in corneal epithelial cells. (**A**) Brightfield images of HCE-T cells at 48 h post-treatment. The appearance of LP-A96 treated HCE-T cells at 48 h was typical of that observed throughout the 3-day incubation period, while all other treatments did not affect the appearance of the cells. Yellow arrows indicate possible perinuclear compartments. (**B**) Cell viability upon 1 μM LP-A96 treatment was quantified alongside other treatments. 50 μM Digitonin was used as a cytotoxic agent. All experiments were performed when cells were subconfluent (30%). At 24 h, LP-A96 vs. A96 treatment: *p* = 0.012*; LP-A96 vs. dPBS: *p* = 0.052. A one-way ANOVA followed by multiple comparisons was used for statistical comparison. * *p* < 0.05. Mean ± SD.

The appearance of cells treated with LP-A96 was also notably different from the cells treated with other agents (Figure 5A) as characterized by the existence of perinuclear refractive organelles (arrows). To confirm that the increase in exosome amount upon LP-A96 treatment was not a result of secreted intracellular vesicles upon cell death, cells were treated with LP-A96 to see if it exhibited any cytotoxicity. During the course of three days, LP-A96 did not appear to exhibit any cytotoxicity (Figure 5B). The proliferation profile of LP-A96 treated cells was similar to that of the cells treated with basal media, Lacripep, or A96. The rate of proliferation was less than that achieved in complete media. At 24 h, the proliferation rate with LP-A96 treatment compared to that with A96 treatment was statistically significant, suggesting a mitogenic effect during the initial 24 h period. However, a mitogenic effect of LP-A96 was not prominent after this period. The difference in cell appearance may be an indirect indication of a change in the intracellular membrane machinery associated with exosome biogenesis and increased vesicular accumulation in multivesicular bodies or other membrane compartments prior to secretion.

Based on the above analyses, we concluded that: (i) the cellular response to exosome biogenesis is more prominent when cell density is low; (ii) purified EVs are exosomes and their increase is dominated by exosomes; and (iii) this increase is not related to the release of membranes upon cell death.

### 2.3. LP-A96 Is Internalized via Dynamin-Mediated Endocytosis and Colocalizes with Syndecan-1 in Corneal Epithelial Cells

**Figure 6:**
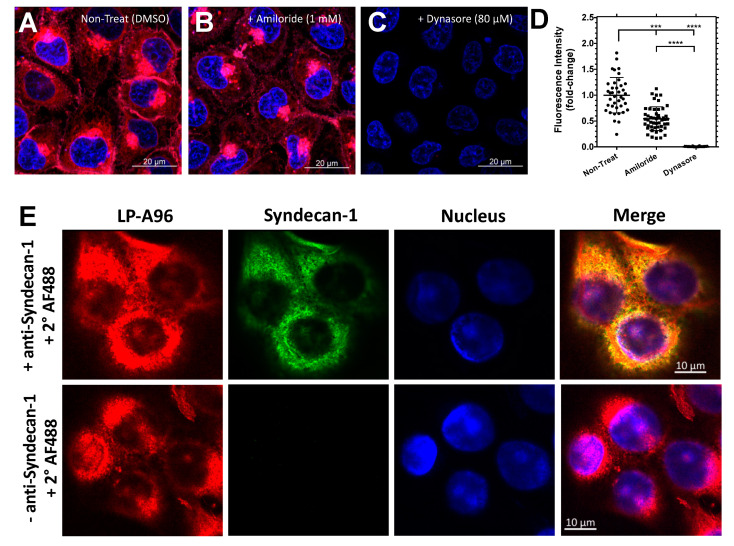
LP-A96 is internalized via dynamin-dependent endocytosis and colocalized with syndecan-1 in corneal epithelial cells. (**A**–**D**) HCE-T cells were treated with either DMSO (vehicle control), amiloride (1 mM), or dynasore (80 μM) prior to addition of 1 μM rhodamine-labeled LP-A96. (**D**) Quantified fluorescence intensity per cell under each treatment. *n* = 39 for DMSO, *n* = 49 for amiloride, *n* = 48 for dynasore. Representative images are shown from the three independent experiments. (**E**) LP-A96 was colocalized with syndecan-1 in HCE-T cells. (Pearson’s correlation = 0.8, *n* = 32). 1 μM rhodamine-labeled LP-A96 were incubated with the cells for 15 min at 37 °C prior to fixation. Secondary immunofluorescence revealed apparent colocalization of rhodamine-labeled LP-A96 and Syndecan-1. Anti-syndecan-1 antibody was further detected by Alexa Fluor 488 conjugated secondary antibody. Nuclei were stained with DAPI (4′,6-diamidino-2-phenylindole). A one-way ANOVA followed by multiple comparisons was used for statistical comparison. **** *p* < 0.0001, *** *p* < 0.001. Mean ± SD.

**Figure A3:**
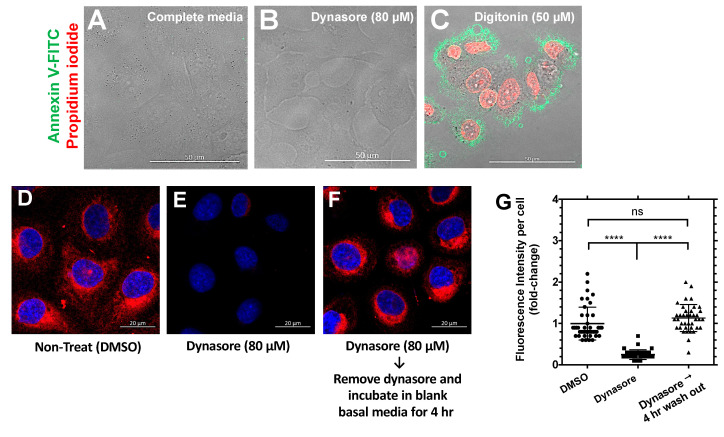
Dynasore inhibits endocytosis of LP-A96 without cytotoxicity. (**A**–**C**) Cell viability under each treatment was visualized using annexin V and propidium iodide. Cells were incubated for 1 h with complete media, 80 μM dynasore, or 50 μM digitonin. Cells treated with dynasore did not experience cell death. (**D**–**F**) Endocytosis of LP-A96 is restored after dynasore has been removed. Cells pre-treated with (**D**) DMSO or (**E**) dynasore were further incubated with 1 μM rhodamine-labeled LP-A96 for 15 min and imaged. (**F**) Cells incubated for additional 4 h in basal media after dynasore was removed were further incubated with 1 μM rhodamine-labeled LP-A96 for 15 min and imaged. (**G**) Fluorescence intensity changes show restoration of LP-A96 endocytosis after 4 h. **** *p* < 0.0001, ns: non-significant. Mean ± SD.

Syndecan-1 is known to be internalized upon clustering at the cell surface [32]. As LP-A96 is expected to cluster syndecan-1 on the cell surface and induce its endocytosis, internalization and subcellular colocalization with syndecan-1 would be expected. First, the internalization of LP-A96 was explored. Cells were treated with either amiloride or dynasore before the addition of LP-A96, which respectively inhibit pinocytosis or receptor-mediated endocytosis [33]. After 15 min of incubation, a significant fraction of LP-A96 is internalized (Figure 6A), partly in an amiloride (Figure 6B) and completely in a dynasore-dependent manner (Figure 6C,D). Dynasore treatment did not affect cell viability and inhibition of LP-A96 endocytosis was restored after dynasore was removed (Appendix A Figure A3). Further confirmation was sought to observe if internalized LP-A96 colocalize with syndecan-1. After 15 min of incubation, LP-A96 colocalized intracellularly with syndecan-1 in the vicinity of the nucleus (Figure 6E). Therefore, it is highly likely that LP-A96 is internalized through a receptor-mediated pathway associated with syndecan-1.

### 2.4. LP-A96 Delivered by Contact Lens Enhances Cell Motility in Corneal Epithelial Cells

**Figure 7:**
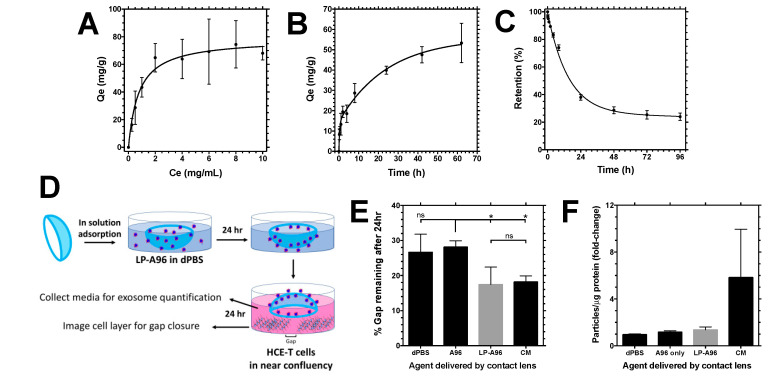
Contact lens-delivered LP-A96 maintains its activity in corneal epithelial cells. (**A**) Concentration-dependent adsorption of LP-A96 to contact lenses over a 24 hr period. (**B**) Time-dependent adsorption of 2 mg/mL LP-A96 to contact lenses. (**C**) Time-dependent release of LP-A96 from contact lenses. (**D**) Study design of the contact lens-mediated delivery of LP-A96 to a scratch (gap) generated corneal epithelial cell monolayer. (**E**) Gap closure activity of HCE-T cells incubated with contact lenses loaded with different agents. (**F**) After gap closure activity measurement, exosomes were purified from culture media and quantified using nanoparticle tracking analyzer. (**F**) Exosome abundance in the culture media after 24 hr period. A one-way ANOVA followed by multiple comparisons was used for statistical comparison. CM indicates complete media. * *p* < 0.05, ns: non-significant. Mean ± SD.

Eyedrops are the most convenient and frequently used route of administration for ocular surface drug delivery. Although it is convenient and effective, a frequent need for topical administration is associated with lower patient compliance. Thus, strategies to enable sustained release of drugs that are effective when given topically are of great interest. Such strategies may maintain an active drug on the ocular surface for a longer period of time. One such method is to use contact lenses as a drug depot. Contact lenses have been studied as a therapeutic platform to manage ocular anterior segment disorders beyond their primary function of providing millions of people with glasses-free vision correction [34,35]. Based on the previous demonstration of adsorption of ELPs to and their delivery to the corneal epithelial cells upon release from the commercially available contact lenses [18], the ability of contact lenses to deliver functional LP-A96 to corneal epithelial cells was tested. To begin with, the adsorption and release kinetics of LP-A96 in contact lenses was determined using a fluorescence-based method. Fluorescein-labeled LP-A96 was incubated with contact lenses to analyze the concentration-dependent adsorption (Figure 7A), time-dependent adsorption (Figure 7B), and time-dependent release kinetics (Figure 7C). Parameters estimated from the fit are reported in Table 2. 

Based on these parameters, contact lenses were incubated with 0.4 mg of LP-A96 for 24 h; release from lenses adsorbed under this condition was expected to increase LP-A96 concentration in culture media up to 1~2 μM over 24 h. After in solution adsorption, LP-A96 loaded contact lenses were transferred to HCE-T cell cultures and incubated for 24 h. Just before the contact lens transfer, a scratch was generated on HCE-T monolayers (Figure 7D). The cells incubated with LP-A96 loaded contact lenses showed equipotent cell motility enhancement to the cells incubated with an intact contact lens in complete media (Figure 7E), which was significantly higher than that observed with A96 loaded contact lens treatment. However, exosome production during this 24 h period was not significantly different (Figure 7F). This may be because LP-A96-mediated exosome biogenesis is not prominent under conditions of maximum HCE-T cell confluency (Figure 3A) and the duration of incubation was only 24 h. Nevertheless, the data clearly show that the contact lenses can deliver functional LP-A96 to corneal epithelial cells.

## 3. Discussion

This study describes the construction of a multivalent Lacritin-derived peptide nanoparticle named LP-A96 and its biological effects in corneal epithelial cells. Under different conditions in corneal epithelial cells, LP-A96 enhanced cell motility and stimulated exosome biogenesis in parallel with intracellular Ca^2+^ mobilization. Of these functions, only the ability to enhance cell motility is shared with the monovalent Lacripep, while the stimulation of exosome biogenesis was unique to the multivalent nanoparticle. When cells are sparse and not in contact with each other, LP-A96 appears to be able to direct the intracellular machinery to evoke exosome biogenesis instead of cell motility. On the other hand, LP-A96 did not activate exosome biogenesis when the cells were confluent but were capable of enhancing cell motility. This cell density-dependent differential activation can be explained by the activation of different cellular mechanism that ensures cell-to-cell communication. When cells are confluent, there is no need for cells to initiate another intercellular communication mechanism, i.e., stimulate production and release of extracellular vesicles for their communication, because cells are able to directly communicate through physical contacts. However, when cells are sparse, the most effective way to communicate with each other would be to stimulate production and release of extracellular vesicles including exosomes containing signaling mediators which can be internalized. This study only tested these two opposing conditions, near-maximal confluency and low cell density. Future studies identifying the conditions under which cells become unresponsive to signaling to evoke exosome biogenesis would allow a better understanding of the syndecan-1 biology in the corneal epithelium.

**Figure A4:**
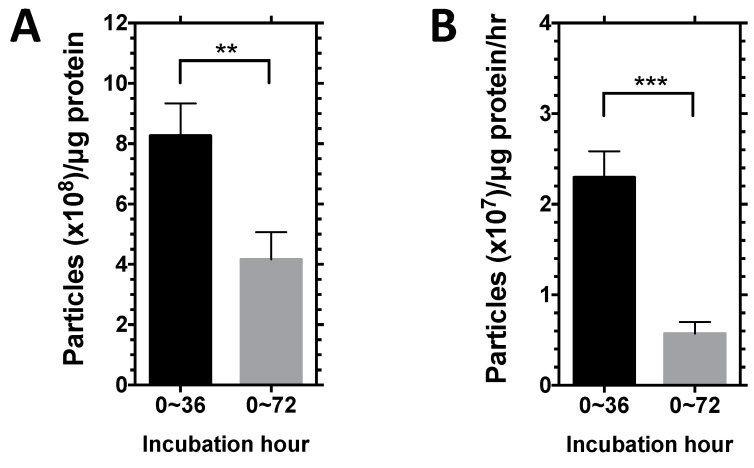
Time-dependent differential release rate of exosomes from HCE-T cells. (**A**) Exosome amounts in culture media were quantified and (**B**) the rate of release was calculated at 36 and 72 h post-treatment. HCE-T cells were treated with 1 μM LP-A96. The total amount of exosomes and the rates of exosome biogenesis/release were higher during the 0~36 h period compared to 0~72 h period. This suggests the rate of exosome uptake/turnover exceeds the rate of exosome biogenesis/release during 36~72 h period as cells reach confluence. *** *p* < 0.001, ** *p* < 0.01. Mean ± SD.

With this, several aspects should also be explored regarding ocular surface physiology. First, LP-A96 treatment stimulated exosome biogenesis/release during the three-day period (Figure 3B). Within a three-day period, the amount of total exosome and the rate of exosome biogenesis/release was higher during the 0~36 h period compared to those of the 0~72 h period (Appendix A Figure A4). This indicates that the exosome uptake/turnover rate may exceed the exosome biogenesis/release rate during the 36~72 h period. As this study focuses on the discovery of the enhancement of exosome biogenesis upon LP-A96 treatment, biogenesis-release-uptake and other biological aspects of ocular exosomes in corneal epithelial cells will be explored in future studies. Second, since LP-A96 stimulates exosome biogenesis, it is possible that the expression and activity profiles of heparinase and proheparanase may change upon addition of LP-A96. As this enzyme is known to be heavily involved in homeostasis [16], a more comprehensive examination of its expression and activity upon LP-A96 treatment in corneal epithelium will allow better insights regarding the biological influence of LP-A96. Third, the involvement of ocular exosomes in corneal wound healing is well documented [36,37]. As a significantly higher number of exosomes that carry RNAs are produced and secreted upon LP-A96 treatment, deep sequencing of miRNAs might shed insights regarding the molecular mechanisms targeted on the ocular surface and possibly the draining lymph nodes by LP-A96-evoked exosomal shedding. Fourth, LP-A96 was colocalized with syndecan-1 in corneal epithelial cells, possibly via association of LP-A96 with syndecan-1 [14]. Further studies addressing direct binding of LP-A96 to syndecan-1 and to other proposed co-receptors, such as G-protein coupled receptors (GPCRs) [12], and their internalization/activation in corneal epithelial cells will be of great importance to understand Lacritin biology at the ocular surface.

The optimal concentration for Lacritin’s mitogenic activity is reported to be 1~10 nM in human salivary gland ductal cells [12]. At this concentration, however, neither Lacripep nor LP-A96 enhanced cell motility in corneal epithelial cells. The chosen concentration for this study was 1 μM, for both Lacripep and LP-A96, based on a dose-dependent scratch closure assay (Figure 2E). As similar concentrations were reported for corneal-lacrimal gland axis in rabbit (0.8~8 μM) [10], mouse (4 μM) [11], and monkey (0.1~1 μM) [9] models, corneal epithelium seems to require a higher dose of Lacritin or its derivative Lacripep compared to salivary gland ductal cells. At 1 μM, only LP-A96 induced Ca^2+^ mobilization but Lacripep did not. Although Lacritin (3.2 nM) was able to evoke Ca^2+^ influx in HCE-T cells [3], it is possible that the structural differences between the full-length versus derivatized peptide fragment affect the binding affinity as well as the degree of Ca^2+^ mobilization at this lower dose. Thus Lacripep-mediated Ca^2+^ mobilization may be transient or under the limits of detection. Another possibility would be that the chosen dose (1 μM) is sub-optimal to detect significant changes based on the ‘bell-shaped’ dose-response curve observed in the parent molecule. Lacritin. Since the current study was conducted with a single dose (1 μM) and is focused on highlighting differences between the free peptide and its multimeric form in eliciting exosome biogenesis, further studies will be needed to define the dose-dependency between Lacripep and Lacritin with respect to both cell motility and calcium signaling. 

In addition to actions on the cornea which have been described, Lacritin is a known tear secretagogue. Upon expression and secretion from LGAC (lacrimal gland acinar cells), Lacritin promotes tear secretion from lacrimal glands to sustain the tear film and maintain ocular surface homeostasis [10]. Upon the development of the LP-A96, its secretagogue activity was tested in primary rabbit LGAC. However, LP-A96 did not show any meaningful secretagogue activity. For this reason, our research efforts were focused on elucidating biological effects in corneal epithelial cells rather than in LGAC. Despite several observational reports of Lacritin’s secretagogue activity, the intracellular mechanisms that promote increased tear secretion are not well understood. Comparison of Lacritin’s secretagogue activity to the well-characterized cholinergic agonist, carbachol, in primary monkey LGACs by Fujii et al. showed that Lacritin stimulated tear secretion in a Ca^2+^ and PKCα independent manner while carbachol required both for its activity [9]. Based on the fact that Ca^2+^ and PKCα are essential molecules for Lacritin-mediated signaling in other epithelial cells, Lacritin’s receptor and intracellular signaling pathway in LGACs seem to be different from what is generally accepted in corneal epithelial cells. Identification of its receptor(s) and its respective intracellular signaling as well as how LP-A96′s multivalency may impact tear secretion, the spectrum of proteins secreted, and exosome production in LGAC will provide more information on how LP-A96 may be used therapeutically in lacrimal glands.

Multivalency was achieved by genetically fusing LP to ELP recombinant polypeptides. Compared to unimeric ELP A96, the hydrodynamic radius of LP-A96 indicate that the amphipathic peptide, LP, drives nano-assembly of LP-A96. Similar assembly by another amphipathic peptide-ELP fusion named L4F-A192 was reported previously [38]. Cryo-TEM imaging revealed that L4F-A192 forms a vesicular structure with a wall thickness of 8.4 nm. Based on the similar amphipathic nature between LP and L4F peptides, it is possible that LP-A96 nano-assembly may form vesicles. Investigating the nanostructures formed by LP-A96 as well as other amphipathic peptide-ELP fusions will allow a better understanding of polymer-mediated nano-assembly.

Drugs that are currently prescribed for the treatment of various ocular surface and anterior chamber disorders have been investigated for contact lens-based delivery to enhance their therapeutic performance. These include drugs for ocular infection (Ciprofloxacin), corneal injury (EGF), allergic conjunctivitis (Ketotifen fumarate), dry eye (Re-wetting agents/hyaluronic acid, Cyclosporin A) and glaucoma (Acetozolamide, Timolol) [39]. Despite these advances, it would be desirable to provide a contact lens drug delivery device which is relatively simple in design; which does not require complicated and expensive manufacturing processes; which does not significantly impair or interfere with the patient’s vision; and which would not require a substantial change in the practice patterns of eye physicians and surgeons. As LP-A96 released from the contact lenses was functional, delivery of LP-A96 through contact lenses could serve as an alternative route of administration to improved ocular surface health.

To conclude, this study demonstrates a simple yet effective peptide modality that is capable of stimulating both cell motility and exosome biogenesis in corneal epithelium, mediated through Lacritin biology. As exosomes mediate cellular communication, signaling, and immune modulation, the biological and therapeutic effects of both exosomes and LP-A96 in the context of dry eye diseases (DED) [40,41] will greatly advance our knowledge towards the pathophysiology of DED and aid in providing a better way to improve daily lives of DED patients.

## 4. Materials and Methods

### 4.1. Synthesis, Expression, and Purification of LP-A96 and A96

The pET-25b(+) vector (#69753, Millipore-Sigma, Burlington, MA, USA) was purchased and further modified for ELP fusion cloning [19]. A chemically synthesized oligonucleotide cassette encoding the amino acids MGKQFIENGSEFAQKLLKKFSLWA was ligated to the N-terminus of ELP A96 to generate LP-A96. The resulting fusion plasmids were sequenced, transformed into, and expressed in ClearColi^®^ BL21(DE3) Electrocompetent Cells (#60810, Lucigen, Middleton, WI, USA). Cells were fermented in terrific broth media supplemented with 1 mM NaCl for 24 h at 37 °C without IPTG induction. After centrifugation, 1 g of biomass (cell pellets) was resuspended in a 4 mL of 1:1 mixture of 1-butanol and ethanol (i.e., mixture of 8 mL 1-butanol and 8 mL ethanol was directly added to 4 g of cell pellet) [42]. Cell pellets were resuspended thoroughly by vortexing (10 s) and left under constant agitation at room temperature for 15 min. After transferring to 50 mL conical tubes, the suspension was centrifuged at 4000 rpm for 10 min using a Sorvall RC-3C Plus Centrifuge. Only the organic phase (upper phase) that contains LP-A96 was collected and transferred to a clean 50 mL conical tube with 5 mL Dulbecco’s phosphate-buffered saline (dPBS, without Ca^2+^ and Mg^2+^). The whole solution was placed under a mild focused air stream with constant stirring, and left overnight to passively evaporate organic solvents. Collected samples were then dialyzed against dPBS for 24 h under sink condition to remove residual organic solvents. Purified proteins were sterile filtered (200 nm pore, #PN 4612, Pall Corp., NY, USA) after dialysis and used for subsequent assays. Lacripep was provided by the Laurie Laboratory.

### 4.2. Biophysical Characterization of LP-A96 and A96

The purity of A96 and LP-A96 fusion proteins was analyzed using SDS-PAGE. The molar extinction coefficients (ε) of A96 and LP-A96 were calculated at 1285 and 6970 M^−1^⋅cm^−1^ [43]. Serial dilutions in Edelhoc buffer were prepared, measured and averaged to acquire the best estimate of protein concentration in dPBS using Equation (1) [43,44].

(1) $$C_{protein}=\frac{A280-\left(A330\times 2\right)}{\varepsilon }.$$

The hydrodynamic radius (*R_h_*) at 25 °C and 37 °C was determined using dynamic light scattering (DLS). Proteins in dPBS (50 μL at 10 μM) were loaded onto a 384-well plate followed by layering with two drops of mineral oil to prevent evaporation, and the whole plate was centrifuged for 1× *g* min at 1000 rcf to remove any remaining air bubbles. Triplicate samples were analyzed using a Wyatt Dynapro plate reader and by built-in software DYNAMICS V7 (Wyatt Tech. Co., CA, USA). *R_h_* was measured first at 25 °C and then the temperature was immediately increased to 37 °C, where the second measurement was made. The plate was subsequently incubated at 37 °C for 7 days, while *R_h_* was measured at days 2, 4, and 7 to observe the stability.

Size exclusion chromatography followed by multiangle light scattering (SEC-MALS) was used to determine the radius of gyration (*R_g_*), absolute molecular weight, and oligomeric state of the sample. 10 μM sample in 100 μL dPBS was injected onto a Shodex size exclusion column (KW-803, Showa Denko K.K, Tokyo, Japan) at 0.5 mL/min. The eluents were analyzed on a Helios system (Wyatt Tech. Co., CA, USA) maintained at 25 °C and the data were fit to a Debye model, which best explained the data to determine the *R_g_*.

The transition temperature (*T_t_*) of proteins were obtained using an optical density. The absorbance at 350 nm, *A*, was measured in a DU800 UV-Vis spectrophotometer (Beckman Coulter, CA, United States) under a temperature gradient of 0.5 °C/min. The *T_t_* at each concentration was defined as the temperature at which the maximum first derivative, *dA/dT*, was achieved using Equation (2). The *A_i_* is defined as the absorbance recorded at *T_i_* temperature. The *T_t_* from each concentration was used to plot the phase diagram and fit with Equation (3) to obtain slope, *m*, and intercept *b* (Table A1).

(2) $${\frac{dA}{dT}|}_{i}\;=\;\frac{A_{i+1}-A_{i}}{T_{i+1}-T_{i}}$$

(3) $$T_{t}\;=\;b-m{\;\log}_{10}\left[C_{protein}\right]$$

### 4.3. Cell Culture, Scratch Assay, and Cytotoxicity

Human corneal epithelial SV40-transformed cells (HCE-T, Riken Cell Bank, Japan) were cultured in KSFM media (#17005042, Life Technologies, Rockville, MD, USA) supplemented with bovine pituitary extract (BPE) and epidermal growth factor (EGF) according to the manufacturer’s recommendation (‘cells’ hereafter). Complete media refers to KSFM supplemented with EFG and BPE and basal media refers to KSFM media alone without EGF and BPE. Cells in passage number 4~7 were used for all cell-based assays. For imaging, a Zeiss LSM880 Confocal Microscope (Carl Zeiss AG, Oberkochen, Germany) equipped with Airyscan, was used (‘confocal microscope’ hereafter). For image analysis, ZEN2 Blue Edition software (Carl Zeiss AG, Oberkochen, Germany) was used (‘ZEN2′ hereafter).

For evaluation of cell motility, cells were cultured in 24 well plates. At 80% confluency, cells were cultured with basal media for another 24 h. A scratch was generated on the cell monolayer using a 200 μL pipette tip. After washing twice with dPBS, cells were cultured with 2 mL of fresh basal media supplemented with 1 μM Lacripep, A96, or LP-A96. These doses were chosen from preliminary studies evaluating optimal dosages of Lacripep and were kept constant in subsequent functional assays. The area that was devoid of cells was imaged under the confocal microscope at 0 and 13 h post-treatment and analyzed using ZEN2. Cells treated with basal media and complete media served as negative and positive controls, respectively. Identical sets of HCE-T cells were also prepared to measure cell motility upon contact lens-mediated delivery of LP-A96. Contact lenses incubated with 400 μg of either LP-A96 or A96 in dPBS (or 10 μM in 1 mL) for 24 h at room temperature under constant agitation were briefly washed in dPBS and transferred to cultures. After 24 h incubation at 37 °C, the cell monolayer was imaged to measure cell motility and cell culture media was collected and processed for exosome purification.

For cell cytotoxicity/proliferation upon LP-A96 treatment, cells were seeded with basal media at 0.1 x 10^4^ cells/well in 96-well plate one day prior to the experiment. On the next day, cells were washed with dPBS and incubated with 1 μM Lacripep, A96, or LP-A96 in basal media. 50 μM Digitonin (#D141, Sigma-Aldrich, St. Louis, MO, USA) was used as a cytotoxic agent. At 24, 48, and 72 h post-treatment, cell proliferation was measured using WST-1 reagent (#5015944001, Sigma-Aldrich, St. Louis, MO, USA) per manufacturer’s protocol.

### 4.4. Confocal Fluoresence Imaging

For calcium signaling, cells at 50% confluency in 35 mm glass-bottom culture dishes were further cultured with basal media for 24 h. Cells were rinsed with dPBS and incubated at room temperature for 20 min with fresh basal media supplemented with 2.5 μM Fluo-4 AM (#F14201, Invitrogen, Carlsbad, CA, USA). After this period, the NaCl Ringer buffer (145 mM NaCl, 5 mM KCl, 1 mM CaCl_2_, 1 mM KH_2_PO_4_, 1 mM MgCl_2_, 10 mM glucose, and 10 mM HEPES, osmolarity 300, pH 7.4) was used to rinse and incubate cells at room temperature for another 30 min. After this period, NaCl Ringer buffer was changed to Ca^2+^ deprived NaCl Ringer buffer (1 mM Ca^2+^ was replaced with 0.5 mM EGTA) and incubated for 10 min. After this period, cells were excited at 488 nm and their emission was recorded in real-time at 510 nm under the confocal microscope. The fluorescent intensity profile was recorded upon addition of 1 μM Lacripep, A96, or LP-A96. The fluorescence profile from each cell was converted to fold-change using Equation (2). *F_0_* is the average fluorescence intensity measured during the first 5 min (before the addition of the treatment) and *F_t_* is the measured fluorescence intensity at every sec.

(4) $$Fold\_change=\frac{\left(F_{t}-F_{0}\right)}{F_{0}}.$$

To observe cellular uptake of LP-A96, cells were cultured in 35 mm glass-bottom culture dishes (#P35G-0-10-C, MatTek Corp. Ashland, MA, USA) in 1.2 mL of basal media supplemented with 10 μL of either DMSO, 1 mM amiloride (#A7410, Sigma-Aldrich, St. Louis, MO, USA), or 80 μM dynasore (#D7693, Sigma-Aldrich, St. Louis, MO, USA) for 30 min at 37 °C. Cells were washed with dPBS twice and then incubated at 37 °C for 10 min with 50 μL of solution that was comprised of 1 μL of NucBlue^TM^ Live Cell Stain ReadyProbes^TM^ reagent (#R37605, Molecular Probes, Eugene, OR, USA), 0.5 μL of rhodamine-labeled LP-A96 (1 μM final concentration), and 48.5 μL of basal media. After a 10 min incubation period, cells were washed with dPBS twice and incubated with Live Cell Imaging Solution (#A14291DJ, Molecular Probes, Eugene, OR, USA). The fluorescence was imaged under the confocal microscope and the intensity was analyzed using ZEN2.

To assay for cell viability upon dynasore treatment, cells cultured in 35 mm glass-bottom culture dishes with either complete medium, 80 μM dynasore, or 50 μM digitonin for 1 h were incubated with annexin V-FITC and propidium iodide (#V13242, Thermo Fisher, Waltham, MA, USA) and imaged per manufacturer’s recommendation.

To colocalize LP-A96 and syndecan-1, 1 μM rhodamine-labeled LP-A96 in 400 μL basal media was incubated with cells at 37 °C for 20 min and fixed/permeabilized with ice-cold methanol:acetone (1:1) mixture for 10 min at −20 °C, and then processed for blocking (1% BSA), anti-Syndecan-1 antibody incubation (1:30 dilution, #SAB1305542, Sigma-Aldrich, St. Louis, MO, USA), and secondary antibody incubation (1:200 dilution, #A21202, Invitrogen, NY, USA). Nuclei were stained with DAPI solution (1:500 dilution, #62248, Thermo Fisher, Waltham, MA, USA) during secondary antibody incubation. Cells were imaged using a confocal microscope and analyzed with ZEN2.

### 4.5. Exosome Purification and Analysis

Cells were seeded into 12-well plates at a density of 0.5 × 10^5^ cells/well. 1 μM of either Lacripep, A96, or LP-A96 were added during seeding. Cells seeded with dPBS or complete medium served as negative and positive controls, respectively. Cells were then incubated at 37 °C for 72 h, undisturbed. After this period, culture media were collected and subjected to exosome purification. After removal of culture media, cells were immediately collected in 50 μL radioimmunoprecipitation assay (RIPA) buffer supplemented with protease inhibitor cocktail (#78430, Thermo Fisher, Waltham, MA, USA) to measure total protein concentration.

To purify exosomes, collected culture media under each condition was cleared of cell debris and microparticles. To do this, the media was spun down at 300 *g* for 5 min. Collected supernatant after this centrifugation was then spun down at 2000× *g* for 10 min. Collected supernatant after this centrifugation was then spun down at 10,000× *g* for 30 min. Collected supernatants after these three centrifugations were concentrated and subjected to column purification (#qEVoriginal/70 nm, iZon Sciences, Medford, MA, USA) that is optimized for exosome purification. The purified exosomes were analyzed for the amount and the size using ZetaView^®^ nanoparticle tracking analyzer (PMX-120, Particle Metrix GmbH, Meerbusch, Germany). The number of exosomes was normalized to the protein concentration in cell lysates measured using the Micro BCA™ Protein Assay Kit (#23235, Thermo Fisher, Waltham, MA, USA). Exosomes in 0.1X dPBS were used for zeta potential analysis in ZetaView^®^.

Exosomes were resolved by SDS-PAGE and blotted with antibodies to CD9 (1:250 dilution, #MA1-80307, Invitrogen, NY, USA), TSG101 (1:500 dilution, #ab3071, Abcam, Cambridge, MA, USA) and Alix (1:500 dilution, #2171, Cell Signaling Technology, Danvers, MA, USA). Primary antibodies were incubated overnight at 4 °C. Donkey anti-mouse (#925-68072, 1:5000 dilution) and goat anti-rabbit (#925-32211, 1:5000 dilution) secondary antibodies were purchased from LI-COR (Lincoln, NE, USA) for fluorescence imaging.

Exosomal RNAs were isolated using miRNeasy Serum/Plasma Kit (#217184, Qiagen, Hilden, Germany) and analyzed with 2100 Bioanalyzer system (Agilent Technologies, Santa Clara, CA, USA).

### 4.6. Adsorption and Release Kinetics of LP-A96 from Contact Lenses

Commercially available Proclear^TM^ 1 Day disposable contact lenses (CooperVision, Inc., Lake Forest, CA, USA) were washed three times with dPBS prior to any studies. To measure the concentration-dependent adsorption of LP-A96 to the contact lenses, excised contact lens pieces (5 mm × 5 mm, about 1~2 mg in weight) were incubated with 100 μL of fluorescein-labeled LP-A96 (#46410, Thermo Fisher, Waltham, MA, USA) for 24 h. After the incubation, contact lens pieces were gently and briefly washed three times in dPBS to remove any unbound material and the fluorescence intensity was measured in 96-well plate using Synergy H1 Hybrid Multi-Mode Reader (BioTek Instruments, Inc., VT, USA). The data were fit with the Langmuir isotherm model (Equation (3)). The *Q_e_* (mg/g) and *C_e_* (mg/mL) are the amount of adsorbed protein per gram of contact lens and protein concentration at equilibrium, respectively. The *Q_m_* (mg/g) is the maximum amount of protein adsorbed per gram of contact lens. The *K_d_* (mg/L) is an equilibrium binding constant.

(5) $$Q_{e}\;=\;\frac{C_{e}\;\times \;Q_{m}}{C_{e}\;+\;K_{d}}.$$

To measure the time-dependent adsorption of LP-A96 to the contact lens, the fluorescence intensity of excised contact lens pieces that were incubated in 100 μL LP-A96 (2 mg/mL) was retrieved at pre-determined time points, gently and briefly washed three times in dPBS to remove any unbound material, and then measured in 96-well plate using Synergy H1 Hybrid Multi-Mode Reader. The acquired data were fit with the two-phase association model.

To determine the release profile of LP-A96 from the contact lenses, intact contact lenses were incubated with 1 mL of fluorescein-labeled LP-A96 (50 μM) overnight at room temperature in a 24-well plate. On the next day, the contact lens was gently and briefly washed three times in dPBS to remove any unbound material and then placed in 4 mL dPBS (pH 7.4) inside a 50 mL conical tube under constant agitation (70 rpm, 37 °C). During the first 24 h, 100 μL dPBS was sampled at predetermined intervals. The dPBS was replaced completely with fresh dPBS after 24 h and for every 24 h thereafter. Collected samples were stored at 4 °C until further analysis. The amount of fluorescein released into the dPBS and the remaining fluorescence intensity on the contact lens after 96 h were measured using a Synergy H1 Hybrid Multi-Mode Reader (BioTek Instruments, Inc., Winooski, VT, USA; Ex: 485 nm/Em: 528 nm). The acquired data were fit the two-phase dissociation model.

## Figures and Tables

**Table 1:** Molecular information of LP-A96, A96, and Lacripep.

Proteins	Amino Acid Sequence	MW (kDa)	
		Expected *^a^*	Measured *^b^*
LP-A96	MGKQFIENGSEFAQKLLKKFSLWAG(VPGAG)_96_Y	39.6	39.5
A96	MG(VPGAG)_96_Y	37.0	37.0
Lacripep	KQFIENGSEFAQKLLKKFS	2.2	2.1

*^a^* Calculated based on the indicated amino acid composition. *^b^* Exact MW of LP-A96, A96, and Lacripep were determined by MALDI-TOF-MS (Appendix A Figure A1D), reported previously [19], and provided by the Laurie Laboratory, respectively.

**Table 2:** Adsorption and release kinetics of LP-A96 in Proclear Compatibles^TM^ contact lenses.

Parameters	Concentration- Dependent Adsorption (Mean (95% CI))	Parameters	Time-Dependent Adsorption (Mean (95% CI))	Parameters	Time-DependentRelease (Mean (95% CI))
*Q_m_* (mg/g)	78.6 (68.5–88.8)	Plateau (mg/g)	55.8 (46.1–65.5)	Fast Release (%)	73.3 (65.8–80.9)
*K_d_* (mg/L)	0.78 (0.35–1.21)	Fast binding (%)	25.6 (15.8–35.4)	*k_fast_* (h^−1^)	0.068 (0.055–0.082)
*R* ^2^	0.84	*k_fast_* (h^−1^)	2.36 (0.0–4.86)	*k_slow_* (h^−1^)	0.0011 (0.0–0.0044)
		*k_slow_* (h^−1^)	0.041 (0.0–0.069)	*t_1/2, fast_* (h)	10.1 (8.4–12.7)
		*t_1/2, fast_* (h)	0.29 (0.14-Inf.)	*t_1/2, slow_* (h)	645.5 (157.3-Inf.)
		*t_1/2, slow_* (h)	16.9 (10.1–51.1)	*R* ^2^	0.99
		*R* ^2^	0.95		

## Appendix A

**Table A1:** Parameters of LP-A96 and A96 phase separation.

Proteins	Phase Diagram *^a^*	
	Slope, m [°C/log_10_(μM)] (Mean [95% CI])	y-intercept, *b* [°C] (Mean [95% CI])
LP-A96	−8.2 [−10.7–−5.7]	58.3 [55.3–61.3]
A96	−21.7 [−29.0–−14.4]	119.5 [105.9–133.2]

*^a^* Fit according to Equation (3).
